# Maternal glucocorticoids have persistent effects on offspring social phenotype irrespective of opportunity for social buffering

**DOI:** 10.1111/1365-2656.70121

**Published:** 2025-09-15

**Authors:** Kirsty J. MacLeod, Alix Bouffet‐Halle, Erik Wapstra, Tobias Uller, Geoffrey M. While

**Affiliations:** ^1^ School of Environmental and Natural Sciences Bangor University Bangor UK; ^2^ Department of Biology Lund University Lund Sweden; ^3^ School of Natural Sciences University of Tasmania Hobart Australia

**Keywords:** intergenerational, maternal effects, maternal stress, social behaviour

## Abstract

1. Exposure to stressors and associated hormones during development can significantly affect offspring phenotype, including social and philopatric behaviour, but these effects can be mediated by the postnatal social environment (‘social buffering’). While the effects of social buffering are well established for complex social behaviours—such as parental provisioning, grooming or cooperative care—the role of social buffering for simpler social interactions—such as parental tolerance of offspring—remains less understood.
2. Here we used the facultatively social viviparous lizard, *Liopholis whitii*, to test the following: (i) the effects of elevated maternal glucocorticoid levels during gestation on offspring mass, growth, dispersal and social interactions after birth; and (ii) whether these effects are mediated by postnatal mother–offspring association.
3. We conducted a factorial experiment in which pregnant lizards were given thrice‐weekly doses of a glucocorticoid hormone (corticosterone) or a control during gestation. Their offspring were then raised either alone or with their mother for 3 weeks. We subsequently released mothers and offspring in large semi‐natural enclosures and quantified offspring mass and social/exploratory behaviour.
4. There were persistent negative effects of prenatal glucocorticoid exposure on offspring growth. We also observed lasting effects of prenatal glucocorticoid exposure on social behaviour: offspring from glucocorticoid‐treated mothers had stronger social associations with other individuals, including with their mother and siblings, compared to offspring from control mothers. Association with their mother early in life did not mediate the effects of prenatal glucocorticoid exposure on offspring phenotype.
5. These effects demonstrate that maternal stress can be an important mediator of variation in social behaviour in lizards, even overriding the influence of the social environment in the early postnatal period. This has potential implications for understanding how social groups form and are maintained.

## Graphical abstract

This study tests whether early‐life maternal association buffers offspring from the effects of prenatal stress in a facultatively social lizard. Despite clear effects of maternal glucocorticoids on growth and social behaviour, social associations did not mitigate these effects, revealing limits to social buffering in this species.

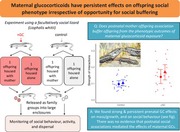

## 1 INTRODUCTION

Maternal effects, the influence of the mother's phenotype on the development of her offspring, can determine as much phenotypic variance within animal populations as do additive genetic effects (Moore et al., 2019). An important source of maternal effects is stressors experienced by mothers. Maternal stress effects may occur through several physiological pathways but have been best studied in the context of the glucocorticoid hormones/the hypothalamic–pituitary–adrenal (HPA) axis (Wingfield, 2013). In brief, when faced with an environmental challenge, activation of the HPA axis and the subsequent release of glucocorticoids is central to organisms' coping and responding appropriately (Wingfield, 2013). However, glucocorticoids in the developmental environment can also alter offspring phenotype and performance with potential implications for population and evolutionary dynamics (Eyck et al., 2019; Harris & Seckl, 2011; Sheriff, 2015).

One way in which developmental stress effects have been suggested to contribute to population and evolutionary dynamics is through effects on social behaviour (Sheriff et al., 2017; Spencer, 2017). Elevated stress can be associated with *activational* (i.e. transiently expressed) effects on social behaviour in adults, such as grouping (Giesing et al., 2011; Muroy et al., 2016) and mate choice (Husak & Moore, 2008). Exposure to stressors during development can also have *organisational* effects (i.e. permanent, determined during development) on the development of social phenotypes, resulting in persistent effects on social behaviours into adulthood. The majority of work to date suggests that exposure to stressors during development makes animals less social (Spencer, 2017). For example, early‐life stress reduces social bonding in prairie voles (*Microtus ochrogaster*: Perkeybile & Bales, 2017), maternal care in rats (*Rattus norvegicus* [Sprague Dawley]: Nephew et al., 2018) and social competence in cichlid fish (*Neolamprologus pulcher*: Reyes‐Contreras et al., 2019). However, these effects are often context‐dependent. For example, zebra finches (*Taeniopygia guttata*) exposed to elevated glucocorticoids during development exhibited reduced foraging associations with their parents in flocks but were more connected to flock members overall than control individuals (Boogert et al., 2014). While these effects have largely been studied in systems with established social structures (e.g. group/family‐living birds and mammals), exposure to elevated stress during development has also been shown to influence social behaviour in species that do not typically exhibit strong social structures. For example, in the common lizard (*Zootoca vivipara*), exposure to prenatal stress promotes philopatry in offspring, increasing the level of association between parents and offspring (De Fraipont et al., 2000; Meylan et al., 2002).

The impact of exposure to maternal stress can be mediated by other aspects of the environment experienced by the offspring (Rossiter, 1998). Positive social interactions can have significant mitigating influences on stress outcomes, a phenomenon termed ‘social buffering’ (Hennessy et al., 2009; Wu, 2021). For example, association with kin or conspecifics reduces stress effects in stickleback (*Gasterosteus aculeatus*: McGhee & Bell, 2014) and in wild macaques (*Macaca sylvanus*: Young et al., 2014). Frequently, social buffering is studied in the context of direct forms of parental care (Gunnar et al., 2015). However, in many lizards, amphibians, fish and invertebrates, parental care is restricted to the tolerance of offspring remaining in natal ranges, with parents rarely interacting directly with their offspring (Rosenblatt & Snowdon, 1996). Whether social buffering plays a role in mediating the stress response when parent–offspring associations are relatively simple is unclear but could contribute to the context‐dependence of social grouping observed in these taxa.

Here, we study maternal stress and postnatal social interactions in a facultatively social viviparous lizard, the White's skink (*Liopholis whitii*), a member of the Egerniinae (hereafter Egernia). Egernia lizards exhibit considerable variation in social behaviour both within and between species, largely characterised by changes in the intensity of interactions between parents and offspring. This variation ranges from species without family ties to facultative tolerance by parents/delayed dispersal of offspring (resulting in the formation of small family groups) to species that live in large, obligate family groups with many (sometimes non‐breeding) adults and multiple cohorts of young (Whiting & While, 2017). In *L. whitii*, parent–offspring associations are relatively simple, extending to offspring remaining in their parent's burrow system (While et al., 2009). The strength of associations between parents and offspring depends on the structure and availability of high‐quality habitat, resulting in variation in the extent of parent‐offspring associations both within and between years (Botterill‐James et al., 2016; Halliwell et al., 2017; While et al., 2009). Experiments in this and closely related species have shown that offspring that are tolerated by their parents gain significant benefits in terms of decreased aggression from conspecifics (O'Connor & Shine, 2004; Sinn et al., 2008) and increased growth rates (Botterill‐James et al., 2016). Further experiments have shown that variation in the postnatal social environment can have strong effects on offspring development in terms of offspring growth, behaviour and learning (Munch et al., 2018; Riley et al., 2017a, 2017b), suggesting the possibility for social buffering.

The aims of this study were to test the following: (i) the effects of maternal stress (mediated by elevated glucocorticoid levels during gestation) on offspring morphology and social behaviour, and (ii) whether these effects on offspring are mediated by mother–offspring association after birth. First, we predict that offspring from females with elevated glucocorticoids during gestation will exhibit reduced social behaviour compared to offspring from control females. Second, we predict that offspring born to mothers with elevated glucocorticoids will be smaller compared to offspring from control females, in line with previous findings across species (e.g. Berghänel et al., 2017). Third, we predict that postnatal association between mothers and offspring should have generally positive effects on offspring based on the assumed benefits of grouping in this species (e.g. Botterill‐James et al., 2016). Therefore, we predict that any negative effects of exposure to elevated maternal glucocorticoids will be reduced by maternal presence in early life in line with the social buffering hypothesis, such that any differences between offspring from glucocorticoid‐treated and control mothers will be reduced or zero.

## 2 METHODS

*Liopholis whitii* is a medium‐sized skink (~75 mm snout‐to‐vent length, hereafter SVL) endemic to south‐eastern Australia. It is distributed across a wide range of habitats and varies geographically in both morphology and life‐history traits (Chapple, 2006). *Liopholis whitii* reaches maturity at approximately 3 years (Chapple, 2006) and is viviparous, possessing a relatively simple chorioallantoic placenta, the likes of which allow for the transfer of signalling molecules, including steroid hormones, between the mother and offspring (as shown in fence lizards: Painter et al., 2002).

### 2.1 Lizard capture and maternal glucocorticoid treatment

Fifty‐seven gravid female *L. whitii* were collected in 2019 from a population at Orford on the east coast of Tasmania, Australia (42°57′ S, 147°88′ E). Captures took place from early November to early December, prioritising female *L. whitii* in the early phase of gestation, assessed using palpation. After capture, lizards were transported to the University of Tasmania's animal facilities where they were individually housed in opaque plastic terraria (57 × 38 × 32 cm). Each enclosure contained a ~5‐cm layer of cat litter as substrate, a rock for basking and a terracotta saucer for refuge. Basking lights (40 W) and overhead UV lighting were set to a day/night cycle (08:00–16:00) to provide thermoregulatory opportunities, while overhead lights were set to a 14:10 hour cycle (light:dark, lights on at 6 a.m.) to approximate light conditions at the capture site. Water was available ad libitum and food (*Tenebrio* larvae) was provided 3× weekly.

Once in the laboratory, females were split into treatment groups: in one group (*N* = 30), females received a 4× weekly transdermal dose of corticosterone (the primary glucocorticoid produced by reptiles) suspended in oil; in the other group (*N* = 27) females received a sham dose of oil vehicle only. Prior to running the experiment, we generated a dose–response curve on an independent sample of lizards to confirm that the glucocorticoid treatment elevated circulating levels of corticosterone within the natural physiological range (see Supporting Information S1 for full details). This treatment simulates the short‐term physiological response to an environmental stressor (the release of glucocorticoid hormones) (Sapolsky, 2002) and has been widely used in studies investigating glucocorticoid effects on lizards (e.g. MacLeod et al., 2018). Females were held under these conditions throughout gestation. Females allocated to the glucocorticoid treatment and control groups did not differ in initial size (SVL, mm) or mass (g) (ANOVA: SVL *F*_1,43_ = 0.38, *p* = 0.54; mass *F*_1,50_ = 0.05, *p* = 0.82). The treatment groups also did not differ in how many doses females received (indicative of length of time spent in treatment before parturition: glucocorticoid group mean = 23 ± 3.6 doses, control group mean = 22 ± 2.8 doses, *χ* ^2^ = 2.68, *p* = 0.10). Nevertheless, we included an interaction of dose number and prenatal treatment in all models to account for possible stronger effects of glucocorticoid treatment if treated for a longer period. As we found no changes to our main effects, we do not report these results in the paper (interaction and main effect results of dose number available in Supporting Information S6).

### 2.2 Early‐life treatment/postnatal social environment manipulation

At the end of gestation (early to mid‐January), terraria were checked twice daily for offspring. Once born, offspring were measured (weight ± 1 mg, SVL ±0.5 mm) and toe‐clipped to allow for unique identification. A total of 60 offspring were born to glucocorticoid‐treated mothers, and 53 to control mothers. One offspring from each litter was removed from the mother and housed singly in small outdoor experimental enclosures (‘solitary’ treatment). Another offspring from the same litter was housed in an identical enclosure but had its mother present (‘family’ treatment). As *L. whitii* give birth to 2–3 offspring on average (but may produce up to five) and offspring are born asynchronously (e.g. spread across several days; While et al., 2007), we used the first two offspring for all subsequent components of the study. Any third and fourth offspring were sacrificed for tissue collection for a separate study or released at the site of capture (*N* = 17). As birth order can have a significant effect on offspring competitive ability (Bouffet‐Halle et al., 2024), we alternated whether the first or second‐born offspring were allocated to the solitary or family treatment across both the glucocorticoid and control treatments. In total, 27 control mothers contributed 22 offspring to the family treatment and 23 to the solitary treatment (19 mothers produced at least 2 offspring each, 7 produced singletons that were distributed across both treatments and 1 female failed to produce any young, resulting in a total of 45 offspring). Thirty glucocorticoid mothers contributed 28 offspring to the family treatment and 23 to the solitary treatment (21 mothers produced at least 2 offspring each, 8 produced singletons that were distributed across both treatments and 1 female produced 2 offspring but one died shortly after birth, resulting in a total of 51 offspring—see Supporting Information S2 for a schematic diagram and details on sample sizes at each time point).

Offspring with or without their mothers were then held in outdoor enclosures for a 3‐week period following birth. This period represents a critical period of postnatal development, during which the social environment can have significant impacts on offspring growth and social behaviour (While & Wapstra, 2008, 2009; see also Munch et al., 2018). Enclosures were 1 m in diameter, covered with bird netting to prevent predation and contained a sandy substrate with rock and brick shelter and natural vegetation to approximate natural conditions. Water was provided ad libitum and food (a handful of *Tenebrio* larvae, with additional provided to the family treatment mother–offspring pairs to ensure that competition over food would not influence offspring) was provided three times weekly. On Day 10 after birth, offspring were temporarily removed from enclosures and measured. All offspring (and their mothers) were removed from these small enclosures at 21 days of age, when they were re‐measured and brought back into the laboratory and prepared for release in large enclosures.

### 2.3 Post‐treatment dispersal/social behaviour assays

To test the interactive effects of maternal stress (elevated corticosterone) and the postnatal social environment (offspring reared alone or with their mother in the first three weeks of life) on offspring activity, social interactions and dispersal, offspring were reunited with their sibling and mother, and then released, with four other family groups, into five large (8 × 16 m) semi‐natural outdoor enclosures at the University of Tasmania's Cambridge Farm facility (see Supporting Information S2 for detailed info and diagram). Each enclosure contained an identical set of evenly distributed resources: ten high‐quality crevice sites (large wooden pallets filled with burrowing substrate [~100 L sand] and topped with cement bricks) and eight supplementary basking sites (lower quality crevice sites consisting of two stacked cement bricks). The enclosures were divided into an 8 × 8 m ‘home’ and ‘dispersal’ section, separated by aluminium flashing with three meshed dispersal ‘gates’ through which offspring (but not adults) could disperse (Halliwell et al., 2017). This design allowed us to create a realistic dispersal scenario where offspring could stay with their mother or move into a vacant territory mimicking the dispersal options open to an offspring in the wild (While et al., 2009). Bird netting was secured over the entire facility to exclude predators. The enclosures contained many natural invertebrates as food and were supplemented with *Tenebrio* larvae three times a week. Water was supplied ad libitum.

The five families (each consisting of one mother with two offspring) were released into the ‘home’ section of one of the enclosures at the same time. Two females without offspring were simultaneously released in the dispersal side of the enclosures to create a more natural dispersal scenario, whereby offspring would be dispersing into areas occupied by other adults. A total of 25 families were released (12 glucocorticoid‐treated mothers and 13 control mothers and their 50 offspring). All individuals were tagged with coloured and numbered bee tags (Pender Beekeping Supplies, Cardiff, Australia) to allow identification using binoculars. Adults were additionally tagged with numbered cloth tape (Tesa SE, Norderstedt, Germany). These markers typically remained in place for 2–3 weeks. Individuals in the enclosures who lost their marker tags were recaptured and the markers re‐applied as soon as possible. Release into enclosures was staggered over a 3.5‐week period (6–27 February 2020) due to the variation in birth dates between females within our sample. Because groups were released as soon as five families were available, the ratio of glucocorticoid‐treated mothers to control mothers was inconsistent between enclosures (E1 and E4 2:3; E2 3:2; E3 1:4; E5 4:1), and there was some variation within each enclosure in the number of days between their time in the small enclosures and the movement into the large enclosures (*N* = 5.24 days on average). We chose this approach as opposed to releasing families into the large enclosures one at a time to ensure that all individuals encountered the enclosure at the same time, as the establishment of territories would be likely to influence the social behaviour and dispersal of offspring released into enclosures after others had already colonised. Five families per enclosure provided a realistic density of lizards. Due to the increasing birth spread towards the end of the breeding season, and because enclosures were fully at capacity, we did not continue to release families after the fifth enclosure was populated.

Lizard positional data (grid coordinates) were collected for 6 weeks for all individuals in each enclosure, which allowed us to generate information on each individual's exploratory activity, dispersal and social interactions. A census of each enclosure was undertaken three times a day (mid‐morning [~9:30–11:00 a.m.], early afternoon [~12–1:30 p.m.] and mid‐afternoon [~2:30–4:30 p.m.]). This involved a researcher walking along the perimeter of the enclosures recording the location of each individual on a digitised enclosure map. Individuals were marked so as to allow identification using binoculars—sufficient distance was maintained to minimise effects on lizard behaviour, following a protocol used in a number of previous studies in this system (Botterill‐James et al., 2016; Halliwell et al., 2017; Moss et al., 2023). Enclosures were monitored in a random order each day. Opportunistic observations throughout the day were also recorded whenever the weather allowed. Lizards that did not change positions more than an hour after initial observation were recorded as new observations. After 6 weeks inside the enclosures (mean age at recap 73.5 ± 4.9 days), all individuals were recaptured and re‐measured before being re‐released at the site of capture. As *L. whitii* reach maturity at around 3 years (Chapple, 2006), the whole experiment was restricted to the early juvenile period. A small number of offspring were not recaptured and were assumed to have died during the enclosure period (*N* = 4: 2 × control + family, 1 × control + solitary, 1 × glucocorticoid + family); for consistency we only included individuals that were recaptured or last seen after the fourth week of the experiment in our analyses.

### 2.4 Quantification of behaviour in the enclosures

#### 2.4.1 Offspring activity

Using the positional data, we extracted the following measures to quantify offspring activity: number of active days (days sighted/total days in enclosure), total number of crevice sites visited, number of crevice site switches and total distance travelled [calculated using the ‘sperich’ package (Lange et al., 2013) in R, which calculates the distance between points in a grid]. We performed a principal component analysis (PCA) on these variables to reduce data dimensionality. Principal components 1 and 2 explained most of the variance (66.7% and 25.6% respectively, Supporting Information S3). Loadings were extracted for each activity variable upon each principal component. Loadings were identified as large when they contributed more than the loadings would be if all variables contributed equally to a given principal component (cut‐off = 0.38). Principal component 1 had strong negative loadings for the total number of crevice sites visited, number of crevice site switches and total distance travelled. Principal component 2 had strong negative loading for the number of active days. These loadings match those using similar data from a previous study of *L. whitii* (Moss et al., 2023) suggesting that these principal components represent consistent and biologically meaningful behaviours in this species. Principal component 3 explained less than 5% of the variance and was not considered further (Supporting Information S3). The PCA suggests that our exploration/activity measures captured two different components of activity: one associated with how many days an individual is seen to be active (PC2) and another which captures *how* active an individual is when seen (PC1). Given this distinction, we use PC1 as our ‘activity’ score and opted to use the raw data for days active rather than PC2 for ease of interpretation (Moss et al., 2023).

#### 2.4.2 Social behaviour

Social interactions were established based on positional co‐occurrence. This is a biologically realistic quantification of social interactions in this system given that parents and offspring that form natural associations do so primarily based on shared crevice/site burrow use and tolerance of offspring (Halliwell et al., 2017; While et al., 2009) with more direct forms of parental care only rarely observed. Individuals A and B were defined as having a social contact if A and B were observed at a location within 1 m distance from each other and the maximum time between these observations of A and B was <60 min (see also Moss et al., 2023). Social contacts between individuals were generated using the R package ‘spatsoc’ (Robitaille et al., 2019). We then used this contact data to construct social networks for offspring and adults within each enclosure using the R package ‘igraph’. This package uses a weighting approach that assigns strength to pairwise associations (equal to the number of interactions divided by total observations of one or both individuals). We extracted the following metrics for each individual from the social networks: number of edges (representing the number of unique individuals a given individual interacts with) and strength of edges (representing the strength of the interactions). We also quantified the strength of dyadic relationships between offspring and their mothers, and offspring and their siblings. We calculated the same metrics for mothers to investigate the potential for treatment during gestation to have continuing effects that may influence offspring social behaviour.

#### 2.4.3 Dispersal

We quantified whether individuals: (i) explored the dispersal section of the enclosures (hereafter referred to as ‘explored’), and (ii) whether they settled in the dispersal section of the enclosures (hereafter referred to as ‘settled’). Individuals were considered to have settled in the dispersal side of the enclosures if they were observed three times sequentially in the dispersal section of the enclosure and were not observed again in the home section (Halliwell et al., 2017). Individuals were scored as a 1 if they explored and/or settled in the dispersal section of the enclosure and a 0 if they did not.

### 2.5 Ethical approval

This study was approved by the University of Tasmania's Animal Ethics Committee (permit number A0018362).

### 2.6 Statistical analysis

We analysed maternal glucocorticoid treatment effects on offspring mass at birth using a general linear mixed model in R (‘lme4’; Bates et al., 2015) with maternal ID as a random effect to account for repeated measures, and maternal treatment as a fixed effect. We fitted mass after birth (i.e. at Days 10, 21 and at recapture) as a response variable in a linear mixed model with maternal and offspring treatment and their interaction as explanatory variables (to test for interacting effects of the maternal glucocorticoid and postnatal social treatments across the juvenile period), and day of measurement (continuous) as additional fixed effects. We included offspring ID, maternal ID and enclosure number as random effects. We additionally tested the effect of treatment on offspring growth (change in mass between birth and recapture per unit time [day]) using the same model structure as above. To estimate effects of our treatments on offspring condition at birth and across the juvenile period, we ran the same models as described above but also included SVL as a fixed effect (i.e. to test effects on body mass, accounting for differences in body size). We did not analyse SVL separately as mass and SVL correlated significantly at all time points (Pearson's correlation, birth: *r* = 0.68, *p* < 0.001; thereafter, *r* = 0.89, *p* < 0.001). We repeated each model to include all offspring born (*N* = 113), just offspring that went into the social environment treatment (2 per mother, *N* = 96), and just those that went into the large enclosure/behavioural assay (*N* = 50). As results were consistent across all data subsets, we report results using the largest sample size (full results in Supporting Information S4).

The effects of maternal and postnatal treatments on offspring activity, social behaviour and binary metrics of exploration/dispersal were analysed using general linear mixed models with the lme4 package (Bates et al., 2015). In all models, maternal glucocorticoid treatment and postnatal social treatment were included as interacting independent variables, and maternal ID and enclosure were included as random terms to account for repeated measures. We also included offspring mass at release (Day 21) in all models to test for potential indirect treatment effects via variation in size; where release mass did not significantly account for variation in behaviour, this was removed from the model and is not reported (results in Supporting Information S5). We also included the number of days sighted (propensity to be active) as an explanatory variable in the model testing our metric of offspring activity as these are likely to be linked.

To allow us to assess to what extent variation in offspring social behaviour was driven solely by offspring, as opposed to or in addition to changes in maternal social behaviour as a result of treatment during gestation, we also fitted two univariate models with maternal social network metrics as response variables, with maternal treatment as a fixed effect. Throughout, count data (number of nodes in social network) were modelled using a Poisson error structure in the same models as described. Binary or proportional dependent variables (days active [days detected of total days in enclosures]; whether offspring explored the dispersal side [0/1] and whether offspring settled in the dispersal side [0/1]) were modelled in the same way but using a binomial error structure and logit link. Social association with siblings is calculated at the family level, so we were not able to test the effects of the postnatal social environment (as both siblings have an identical value of association with each other). This model did not include maternal ID as a random term, as there was only one measure per family.

For models containing interaction terms, p values were calculated using Type III Wald chi‐squared tests. In all models, where interactions were not significant, they were removed, and parameter estimates were calculated from main effects‐only models.

## 3 RESULTS

There were no effects of maternal glucocorticoid treatment on birth spread (Kruskal–Wallis *χ* ^2^ = 0.43, *p* = 0.51), litter size (Kruskal–Wallis *χ* ^2^ = 0.04, *p* = 0.84), litter mass (ANOVA *F*_1,54_ = 0.30, *p* = 0.59) or maternal postpartum mass (*F* ANOVA _1,55_ = 0.82, *p* = 0.37).

### 3.1 Treatment effects on offspring morphology

**FIGURE 1:**
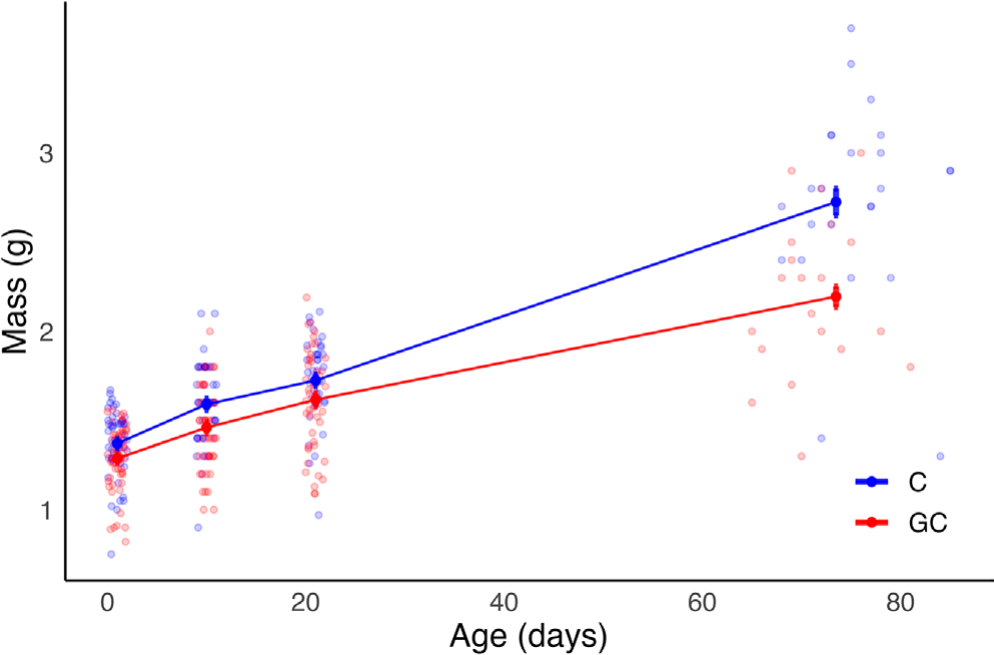
Effect of maternal treatment on offspring mass (g). Offspring were measured at birth, and on Days 10 and 21 before release into enclosures (datapoints from these measurements are jittered to better show data spread). They were then remeasured at recapture, where there was some variation in age due to (i) staggered release into the enclosures and (ii) the fact that recaptures occurred over a period of time (so age at recapture is dependent on when an animal was caught). Data from recapture is not jittered due to natural spread in data. Offspring from glucocorticoid treated mothers are shown in red (GC), those from control mothers in blue (C).

Offspring born to mothers treated with glucocorticoids were significantly lighter at birth than offspring born to control mothers (Table 1a; Figure 1), although this difference was not present when controlling for snout‐to‐vent length (i.e. no statistically significant effect on ‘body condition’; Table 1b). The negative effect of maternal glucocorticoid treatment on offspring mass persisted throughout the experiment (Table 1a(ii); Figure 1). Offspring mass over time was strongly influenced by initial mass but also by differences in growth rate between treatments (Table 1c). Maternal glucocorticoid treatment also significantly negatively affected offspring body condition during this period (Table 1b(ii)). There was no effect of postnatal social treatment on offspring size or condition alone or in interaction with maternal treatment (Table 1a–c; prenatal GC × social treatment: mass [Day 10‐recapture] *χ* ^2^ = 0.04, *p* = 0.84, condition [Day 10‐recapture] *χ* ^2^ = 0.03, *p* = 0.86, growth rate [birth‐recapture] *χ* ^2^ = 0.03, *p* = 0.86).

**TABLE 1:** Effects of maternal glucocorticoid (GC) and postnatal social (solitary, family) treatment on offspring mass and offspring mass controlling for SVL in the model (i.e. testing for an effect of treatment on ‘body condition’).

		Fixed effects	*β*	SE	*T*	*p*	95% CI
(a) Mass	(i) Birth (*N* = 113)	Intercept	1.38	0.03	44.92	<0.01	1.32, 1.44
		Maternal GC treatment (+GC)	−0.09	0.04	−2.14	0.04*	−0.17, −0.01
	(ii) Day 10—recapture (*N* = 89)	Intercept	1.44	0.06	25.45	<0.001	1.33, 1.55
		Maternal GC treatment (+GC)	−0.16	0.06	−2.62	0.009**	−0.29, −0.04
		Social treatment (solitary)	0.0007	0.05	0.14	0.89	−0.10, 0.12
		Age	0.02	0.0006	28.36	<0.001***	0.01, 0.02
(b) Condition (mass controlling for size)	(i) Birth (*N* = 113)	Intercept	−1.03	0.29	−3.62	<0.001	−1.60, −0.47
		Maternal GC treatment (+GC)	−0.05	0.03	−1.57	0.12	−0.11, 0.01
		SVL	0.66	0.08	8.47	<0.001***	0.50, 0.81
(b) Condition (Day 10—recapture)	(ii) Day 10—recapture (*N* = 89)	Intercept	1.51	0.06	24.98	<0.001	1.39, 1.63
		Maternal GC treatment (+GC)	−0.16	0.06	−2.66	0.009**	−0.29, −0.04
		Social treatment (solitary)	0.008	0.05	0.16	0.88	−0.10, 0.11
		Age	0.007	0.002	2.84	0.005**	0.00, 0.01
		SVL	0.01	0.004	2.90	0.004**	0.00, 0.02
(c) Growth rate (mass change per unit time [day])	(i) Birth to recapture (*N* = 44)	Intercept	0.002	0.005	0.37	0.72	−0.01, 0.01
		Maternal GC treatment (+GC)	−0.004	0.002	−2.62	0.01*	−0.01, 0.00
		Social treatment (solitary)	0.002	0.001	1.83	0.08	0.00, 0.00
		Birth mass	0.01	0.004	3.06	0.004**	0.00, 0.02

*Note*: Fixed effects significant at *α* < 0.05 are in bold (* denotes significance at *p* < 0.05, ** denotes significance at *p* < 0.01, and *** denotes significance at *p* < 0.001). In all models, where interactions were not statistically significant, they were removed (see main text for interaction effects).

### 3.2 Post‐treatment behaviour and dispersal

**FIGURE 2:**
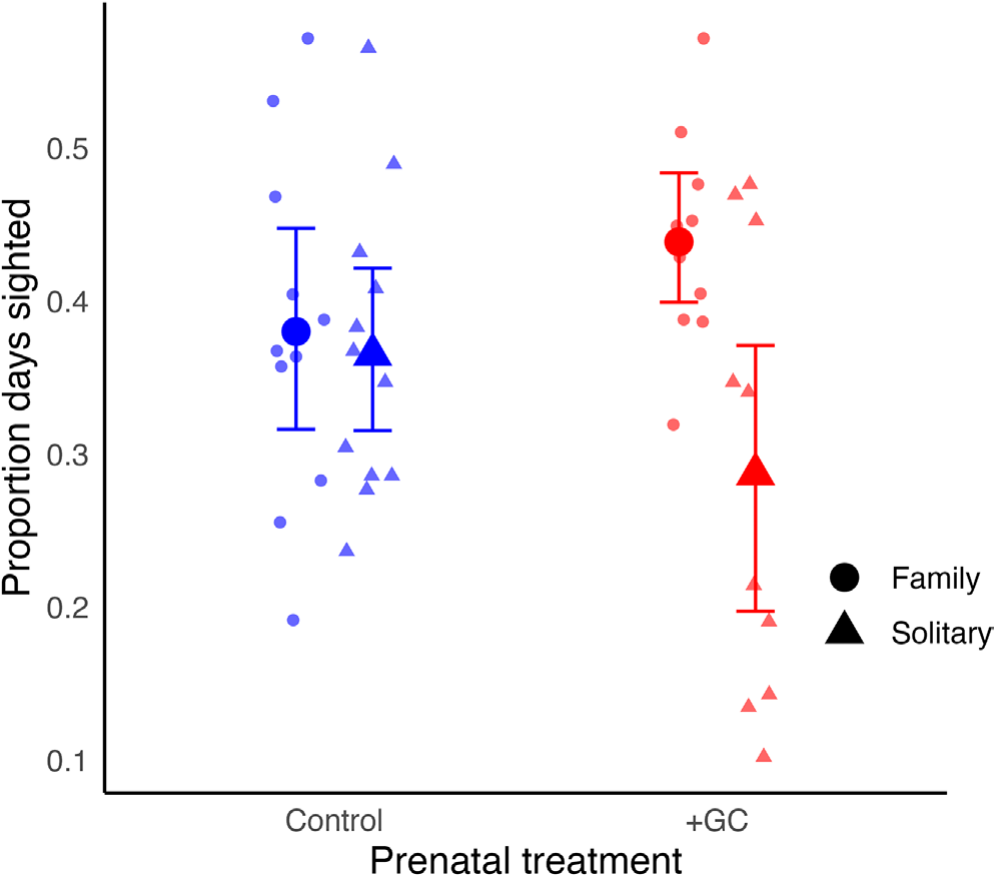
Maternal glucocorticoid (GC/control) treatment and postnatal social treatment effects on the proportion of days offspring were sighted.

A total of 25 females and their offspring (*n* = 50) were released into the outdoor enclosures and monitored for 46.7 ± 3.12 days (range 42–55). During this time, a total of 2791 positional (1478 offspring positions and 1313 adult positions) recordings were made across the five enclosures. Individuals were active (days sighted/total days in enclosures) on average 36.8% of the time (range 10.2%–57.1%). Days active were influenced by an interactive effect of maternal and postnatal treatments (*Z* = −2.31, 95% CI −0.75, −0.06, *p* = 0.02, Figure 2), and by mass at release (positive effect: *Z* = 2.66, 95% CI 0.14, 0.91, *p* = 0.008). Among offspring born to glucocorticoid‐treated mothers, those that stayed with their mothers after birth were more frequently observed than those reared alone (post‐hoc test, estimated marginal means with Tukey adjustment *Z* = 3.61, *p* = 0.002), while among offspring born to control mothers, there was no difference based on postnatal social treatment (*Z* = 0.62, *p* = 0.92). There was no effect of maternal glucocorticoid treatment on our metric of offspring activity (principal component 1 comprising number of crevice site switches, number of unique crevice sites and total distance moved) (*T*_39_ = −0.61, 95% CI −1.16, 0.62, *p* = 0.54). Likewise, there was no effect of postnatal social environment (*T*_39_ = −0.03, 95% CI −0.80, 0.78, *p* = 0.98), or an interaction of maternal and postnatal treatments (*χ* ^2^ = 1.59, *p* = 0.21). Individuals that were sighted more were more active (*T*_39_ = 3.21, 95% CI 0.05, 0.20, *p* = 0.003).

Social network analysis revealed no significant effects of either maternal or postnatal treatment, or an interaction of both, on the number of unique interactions (number of edges) in an offspring's network (Table 2a; prenatal GC × social treatment: *χ* ^2^ = 0.53, *p* = 0.47). However, the overall network score (strength of edges) was significantly higher for offspring born to glucocorticoid‐treated mothers (Table 2b). There was no effect on the strength of social interactions of postnatal social treatment (Table 2b), and no interactive effect (Prenatal GC × social treatment: *χ* ^2^ = 0.58, *p* = 0.45). Similar results were observed when we explored the role that glucocorticoid treatment during gestation had on adult female social network metrics. Specifically, while glucocorticoid treatment during gestation did not influence the number of interactions in a female's network (*Z* = 0.12, 95% CI −0.37, 0.42, *p* = 0.90), glucocorticoid‐treated females had a higher strength of interactions than control females (*T*_21_ = 2.74, 95% CI 0.02, 0.15, *p* = 0.01).

**TABLE 2:** Results from GLMMs testing effects of pre‐ and postnatal treatments on offspring social behaviour.

	Fixed effects	*β*	SE	Test statistic	*p*	95% CI
(a) Number of unique interactions	Intercept	1.34	0.14	9.34	<0.001	1.06, 1.62
	Maternal GC treatment (+GC)	−0.10	0.18	−0.57	0.57	−0.46, 0.25
	Social treatment (solitary)	0.03	0.16	0.17	0.86	−0.28, 0.33
(b) Strength of interactions	Intercept	0.09	0.02	5.45	<0.001	0.06, 0.13
	Maternal GC treatment (+GC)	**0.06**	**0.02**	**2.55**	0.02*	**0.01, 0.10**
	Social treatment (solitary)	0.02	0.01	1.45	0.15	−0.01, 0.05
(c) Association strength between offspring–mother dyads	Intercept	−3.78	0.22	−17.07	<0.001	−4.22, −3.33
	Maternal GC treatment (+GC)	**0.71**	**0.23**	**3.06**	0.004**	**0.24, 1.17**
	Offspring social treatment (solitary)	0.13	0.17	0.77	0.44	−0.21, 0.48
(d) Association strength between sibling dyads	Intercept	−3.86	0.18	−21.08	<0.001	−4.25, −3.47
	Maternal GC treatment (+GC)	**0.68**	**0.25**	**2.69**	0.02*	**0.15, 1.22**

*Note*: Test statistic is *T* except for (a), a generalised linear model which reports *Z*. Fixed effects significant at *α* < 0.05 are in bold (* denotes significance at *p* < 0.05, ** denotes significance at *p* < 0.01, and *** denotes significance at *p* < 0.001). In all models, where interactions were not statistically significant, they were removed (see main text for interaction effects).

We then focused on social associations between offspring and their mother and between siblings (for network visualisations see Supporting Information S5). Offspring from glucocorticoid‐treated mothers had significantly higher network associations with their mothers compared to offspring from control mothers (Table 2c) irrespective of their postnatal social treatment (prenatal GC × social treatment: *χ* ^2^ = 2.23, *p* = 0.14). Siblings from glucocorticoid‐treated mothers also had significantly stronger associations with each other compared to siblings from control mothers (Table 2d). Social association with siblings was calculated at family level (i.e. within sibling dyads) so we were not able to test the effects of the postnatal social environment. Social associations between offspring and their mothers were not significantly affected by the offspring postnatal social treatment (Table 2c).

**FIGURE 3:**
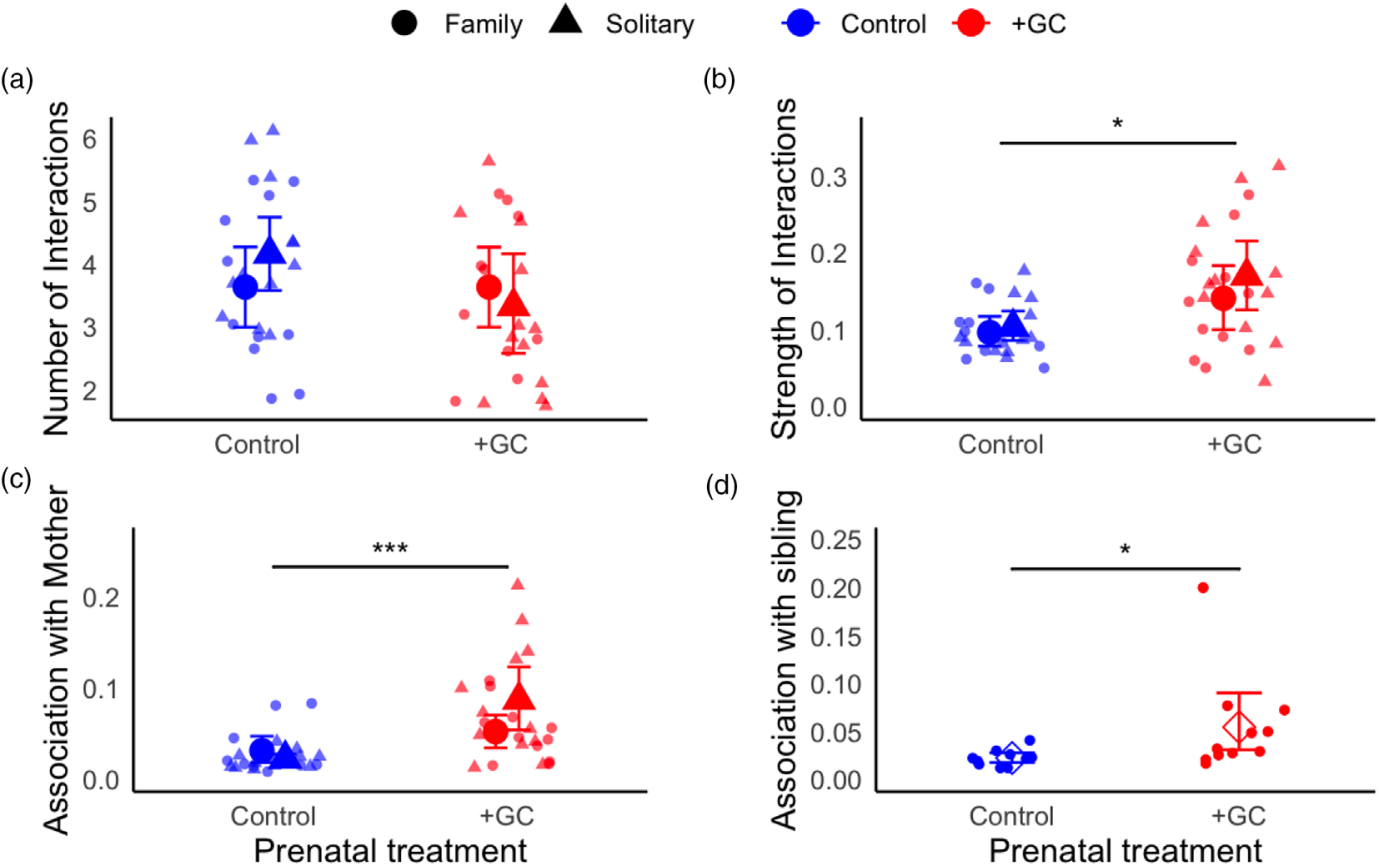
Pre‐and postnatal treatment effects on social network metrics: (a) number of unique interactions; (b) network score (strength of associations); (c) strength of maternal–offspring interactions; (d) strength of sibling interactions. Offspring from GC‐treated mothers in red, control offspring in blue. (* denotes a significant effect at *p* < 0.05; *** denotes a significant effect at *p* < 0.001.)

Exactly half of the surviving offspring (23 out of 46) explored the dispersal section of the enclosures (maternal treatment: 12 control vs. 11 glucocorticoid; postnatal treatment: 8 family vs. 15 solitary). Out of 46 offspring, 11 settled into the dispersal section (maternal treatment: 5 control vs. 6 glucocorticoid; postnatal treatment: 3 family vs. 8 solitary) while the 35 remaining offspring stayed in the home side of the enclosures. There was no significant effect of maternal treatment on whether offspring explored the dispersal section of the enclosures (*Z =* 0.29, 95% CI −1.55, 2.08, *p* = 0.77), or whether they ultimately settled in the dispersal section (*Z =* 0.36, 95% CI −1.59, 2.31, *p* = 0.72). Likewise, there was no significant effect of postnatal social environment on whether the dispersal side was explored (*Z* = 1.53, 95% CI −0.39, 3.19, *p* = 0.13) or whether offspring dispersed (*Z =* 1.50, 95% CI −0.46, 3.46, *p* = 0.13) or any interactive effects of maternal and postnatal treatments (exploration: *χ* ^2^ = 0.01, *p* = 0.90; dispersal: *Z* = 0.37, *p* = 0.54) (Figure 3).

## 4 DISCUSSION

Here we tested whether elevated maternal glucocorticoids influenced offspring growth and the expression of activity, exploration, social behaviour and dispersal. We also tested whether these effects were modulated by the presence of the mother in early life. While we found strong and persistent effects of maternal exposure to corticosterone on offspring growth and social behaviour, we found no evidence that these effects were mediated by the presence of the mother following birth.

Maternal glucocorticoid treatment had strong and persistent effects on offspring social behaviour. While maternal glucocorticoid treatment did not influence the number of individuals an offspring interacted with, it influenced the strength (i.e. frequency) of those interactions. Exposure to stress during development results in reduced social behaviour in many species (Spencer, 2017), but such effects are not always observed. For example, in zebra finches (*Taeniopygia guttata*), individuals treated with glucocorticoids in early life associate with a greater number of conspecifics and are more central in the overall social network in later life (Boogert et al., 2014). Exposure to predation risk, an important ecological stressor, causes female stickleback to produce eggs with higher concentrations of glucocorticoids, resulting in juveniles with higher baseline levels of shoaling behaviour (*Gasterosteus aculeatus*: Giesing et al., 2011). The observed effects of maternal stress on the strength of social associations in our experiment were particularly pertinent in the context of familial interactions, with glucocorticoid‐treated females and their offspring having stronger associations than control mothers with their offspring. This is consistent with experiments in common lizards (*Zootoca vivipara*) that demonstrated that offspring born to glucocorticoid‐treated mothers showed greater attraction to maternal odour and were more philopatric (De Fraipont et al., 2000). Such increased affiliative behaviour and social cohesion in response to signals of stress may be adaptive, for example, if it allows individuals to gain benefits of group living (Spencer, 2017). Indeed, in Egernia lizards such affiliative behaviour between parents and offspring has been shown to have several functional benefits, including reduced conspecific aggression (O'Connor & Shine, 2004; Sinn et al., 2008) and reduced predation risk (Watson et al., 2021).

Contrary to expectations, association with mother early in life did not appear to mediate the effects of glucocorticoid exposure on offspring phenotype. The only effect observed in this experiment was that offspring from glucocorticoid‐treated mothers were active on fewer days if they had been kept isolated from their mother after birth compared to offspring that had been kept with their mother. However, we found no evidence that these individuals were also less social (Table 2), suggesting that the effect primarily concerns general activity levels. This is consistent with previous research, which showed that social isolation influences an offspring's propensity to emerge after a threat (Munch et al., 2018). The stronger effect observed in offspring from glucocorticoid‐treated mothers, but not those from controls, may indicate that stress exposure exacerbates sensitivity to social context. The nature of this effect provides some evidence that the postnatal social environment mediates potentially negative effects of maternal stress on one aspect of individual activity (e.g. the opportunity for exploration). In contrast, we found no evidence that the early social environment mediates the effects of maternal glucocorticoids on offspring social behaviour later in life. One explanation for the weak effect of post‐birth physical association between offspring and mother is that maternal behaviour in this species is not sufficient to have a substantial effect on offspring physiology or cognitive development. Social buffering is often mediated via the triggering of physiological effects that counter stress or reduce reactivity (Wu, 2021). In many species studied to date, the mechanism by which this occurs is through direct interactions linked to maternal care (e.g. the licking‐grooming rodent model; Weaver et al., 2004) which activate neuropeptide secretion that has positive effects on individuals (e.g. Smith & Wang, 2014). Much remains to be understood about the hormonal basis of reptile social interactions (but see Campos & Belkasim, 2021; Lind et al., 2017), specifically in the context of the social factors that govern their release. Postnatal associations in reptiles (including *L. whitii*) tend to be relatively indirect, extending simply to tolerance of offspring within the natal home range (While et al., 2009). While this likely provides some fitness benefit (see above), parental tolerance as opposed to provisioning or care may not be sufficient to counter organisational effects of maternally‐derived glucocorticoids.

In addition to effects on offspring social behaviour, we also observed strong effects of maternal glucocorticoids on offspring mass (offspring from glucocorticoid‐treated mothers were lighter at birth). That elevated glucocorticoids in mothers cause smaller offspring is commonly observed (e.g. Berghänel et al., 2017; Eyck et al., 2019; Sheriff et al., 2009; Zanette et al., 2011), including in lizards (Cadby et al., 2010; Itonaga et al., 2012, but see Ensminger et al., 2018). In the common lizard (*Zootoca vivipara*), size, body condition and growth of offspring from corticosterone‐treated mothers are reduced compared to offspring from control mothers (Meylan & Clobert, 2005). There may be adaptive benefits to smaller body size in response to stressors: for example, smaller body size may confer anti‐predator advantage in predator‐dense sites/periods, meaning that maternal glucocorticoids act as an ecological integrator to adaptively ‘match’ offspring to the prevailing environment (Sheriff & Love, 2013). Potential adaptive benefits related to size in this species are, however, unclear. It has been suggested that such parental effects may weaken over ontogeny (Moore et al., 2019); however, in our study offspring from glucocorticoid‐treated mothers remained smaller than those from control mothers even 2–3 months after birth, despite substantial variation in the postnatal environment. These differences were enforced by changes in growth rate in the postnatal period in addition to and independent of initial mass differences. This suggests that stress‐related maternal effects on morphology can remain throughout early life, perhaps through differential growth mediated by effects on behaviour. For example, growth is partly mediated by the opportunities for basking (Cadby et al., 2014; Wapstra, 2000) and basking behaviour can be mediated by glucocorticoid exposure during early development (e.g. Cree et al., 2003). Our results demonstrate the value of repeated and longer term measurement of glucocorticoid effects in offspring, which remain relatively uncommon (MacLeod et al., 2021).

The White's skink has been well studied with respect to the causes and consequences of social groupings (e.g. Halliwell et al., 2017; While et al., 2009). The effects of maternal glucocorticoids on offspring behaviour, growth and affiliation raise two key questions. First, what are the mechanisms by which maternal stress increases affiliative behaviour? Second, what are the broader consequences of these effects for social groupings in this species? In species with relatively simple social structures such as *L. whitii*, affiliative behaviour is largely driven by philopatry. Therefore, an increased strength of association between mother and offspring could emerge via maternal stress effects on offspring philopatry, as has been observed in other lizard species (e.g. Meylan et al., 2002). However, despite positive effects on social behaviour, we did not find evidence for any strong effect of maternal glucocorticoid treatment on our measures of exploration or dispersal (though there was an interactive effect on days sighted in the GC‐treated group, which could influence opportunities for both). Alternatively, increased social behaviour could be driven by greater affiliative behaviour *per se*. One potential route is via the oxytocin‐family neuropeptides (oxytocin and vasopressin in mammals, and their homologues in other vertebrates, e.g. mesotocin and vasotocin in birds and reptiles) which are strongly linked to social behaviour (Adkins‐Regan, 2009). The HPA axis interacts with nonapeptide systems, such that stress‐induced changes in neuropeptides can have downstream consequences on social behaviour. For example, chickens from corticosterone‐treated eggs show altered stress physiology, reduced arginine vasotocin expression and increased juvenile aggression (*Gallus gallus domesticus*: Ahmed et al., 2014). Finally, since glucocorticoid‐treated mothers had stronger social network connections than controls, stronger affiliation between mothers and offspring may partly be the result of glucocorticoid‐treated mothers increasing tolerance towards offspring in the enclosures. Thus, greater parent‐offspring associations may result from glucocorticoids mediating both offspring and adult behaviour. Disentangling whether these effects are moderated through the female or offspring represents an interesting avenue for further research.

Irrespective of the mechanisms involved, the effect of glucocorticoids in the developmental environment on offspring and parental behaviour has important implications for understanding how social groups form. The increased strength of associations between closely related individuals of gestationally ‘stressed’ mothers potentially provides a mechanistic pathway that connects environmental context (e.g. predation risk, food availability, climatic harshness) to the emergence and evolution of social living via the organisational effects of developmental stress. The role that these environmental stressors play in mediating the emergence of social living is often explored in terms of the functional benefits social grouping provides in dealing with such stressors. However, our results suggest that these effects may have a common mechanistic basis that may increase associations irrespective of the specific function of social grouping. Organisational effects of exposure to stress may therefore mediate both the initial behavioural response (closer social associations) and facilitate longer term retention of an environmental response (i.e. associations can be then selected for where they provide a benefit to either offspring and or parents; Badyaev, 2005). The stress response may therefore provide the foundation from which behavioural responses can be co‐opted and refined, resulting in the evolution of more complex social systems.

## AUTHOR CONTRIBUTIONS

Kirsty J. MacLeod, Geoffrey M. While and Tobias Uller conceived the study; all authors contributed to experimental design and logistics; Kirsty J. MacLeod, Alix Bouffet‐Halle, Erik Wapstra and Geoffrey M. While conducted fieldwork and collected data; Kirsty J. MacLeod and Alix Bouffet‐Halle analysed the data; Kirsty J. MacLeod, Alix Bouffet‐Halle and Geoffrey M. While wrote the manuscript; all authors contributed to editing.

## CONFLICT OF INTEREST STATEMENT

The authors declare no competing interests.

## Supporting information


**Data S1.** **Supporting Information S1.** Glucocorticoid (corticosterone) dose–response test, November 2018.
**Supporting Information S2.** Experimental design.
**Supporting Information S3.** Calculation of activity metric from principal component analysis.
**Supporting Information S4.** All results from morphological analyses including data subsets.
**Supporting Information S5.** Social network visualisations.
**Supporting Information S6.** Effects of maternal dose number, mass at release.
**Figure S1.1.** Circulating corticosterone levels in pregnant *L. whitii* following a CORT dose (0.8 μg corticosterone/g body mass), measured at five timepoints post‐dose.
**Figure S2.1.** Diagram outlining the experimental protocol and sample sizes at each step. Red boxes (left panel) indicate glucocorticoid‐treated mothers/subsequent offspring; white boxes (right panel) indicate control mothers/subsequent offspring.
**Figure S2.2.** Photo of the large semi‐natural outdoor enclosures in Cambridge, Tasmania. Photo by Geoffrey While.
**Figure S2.3.** Diagrammatic representation of one semi‐natural outdoor enclosure in Cambridge, Tasmania. Enclosures each contained an identical set of evenly distributed resources: ten high‐quality crevice sites (large wooden pallets filled with burrowing substrate [~100 L sand] and topped with cement bricks) and eight supplementary basking sites (lower quality crevice sites consisting of two stacked cement bricks).
**Figure S3.1.** Loadings from PCA including total unique pallets, switches between pallets, total distance moved, and number of days ‘out’ (observed).
**Figure S5.1.** Social networks in enclosures 1–5. Each circle represents an individual, circle size is proportional to the number of unique interactions with conspecifics that an individual has and the thickness of the ties between circles represents the strength of interactions between two individuals. Mothers and offspring are identifiable by naming convention (i.e. Mother E1_1, Offspring E1_1‐1, E1_1‐2).

## Data Availability

Data are available at DOI: 10.5281/zenodo.16753038 (MacLeod et al., 2025).
