# Supplementary material for: Maternal glucocorticoids have persistent effects on offspring social phenotype irrespective of opportunity for social buffering
Source: J Anim Ecol. 2025 Sep 15;95(8):1326–38. doi: 10.1111/1365-2656.70121 (PMC13439590; doi:10.1111/1365-2656.70121)
Supplement: Supplementary file 1 [file JANE-95-1326-s001.docx]

**Supplementary info S1** – Glucocorticoid (corticosterone) dose response test, November 2018

Twenty-five pregnant female *L. whitii* were given a CORT dose, administered via pipette onto the dorsal area of the lizard, with the volume corrected for body weight (0.2μL/g lizard of 4 mg corticosterone in 1mL of sesame seed oil vehicle, resulting in doses of 0.8μg corticosterone/g body mass), following the protocol of MacLeod et al. (2018). By altering hormone levels directly, we circumvent any potential between-female variation in perception of stress, thereby maintaining standardization.

To confirm a) that the dose elevated circulating CORT, and b) that the dose elicited only a short-term elevation of CORT (confirmed by circulating levels returning to near-baseline within hours), a blood sample was taken from each female across a range of timepoints (mins post-dose): 0, 30, 60, 90, and 360, with 5 females contributing a blood sample at each timepoint. Blood samples were centrifuged and plasma pipetted off and immediately frozen prior to assay for CORT levels using a commercially available enzyme immunoassay kit following kit instructions (Arbor Assays, Ann Arbor MI, USA).

The dose response curve is shown below (mean group value): the dose rapidly elevated blood corticosterone levels, with CORT levels returning to close to baseline by 6 hours later.

Figure S1.1 Circulating corticosterone levels in pregnant *L. whitii* following a CORT dose (0.8μg corticosterone/g body mass), measured at 5 timepoints post-dose.

**Supplementary information S2 – Experimental design**

Figure S2.1. Diagram outlining the experimental protocol and sample sizes at each step. Red boxes (left panel) indicate glucocorticoid-treated mothers/subsequent offspring; white boxes (right panel) indicate control mothers/subsequent offspring.

Figure S2.2: Photo of the large semi-natural outdoor enclosures in Cambridge, Tasmania. Photo by Geoffrey While.

Figure S2.3: Diagrammatic representation of one semi-natural outdoor enclosure in Cambridge, Tasmania. Enclosures each contained an identical set of evenly distributed resources: ten high-quality crevice sites (large wooden pallets filled with burrowing substrate [~100 L sand] and topped with cement bricks) and eight supplementary basking sites (lower quality crevice sites consisting of two stacked cement bricks).

**Supplementary info S3 –** Calculation of activity metric from Principal Component Analysis

S3.1 Loadings from PCA including total unique pallets, switches between pallets, total distance moved, and number of days “out” (observed).

|  | Dim 1 | Dim 2 | Dim 3 |
| --- | --- | --- | --- |
| Nb_days_out | 0.4082949 | 0.9011506 | 0.10857015 |
| Total_unique_pallets | 0.8748576 | -0.4162876 | 0.03575138 |
| Pallet_switches | 0.9374642 | -0.1391688 | 0.25822236 |
| total_dist | 0.9253101 | 0.1369518 | -0.34332299 |

S3.2 Activity PCA output info including eigen values of each PC.

|  | eigenvalue | percentage of variance | cumulative percentage of variance |
| --- | --- | --- | --- |
| PC 1 | 2.6671187 | 66.677966 | 66.67797 |
| PC 2 | 1.0234915 | 25.587287 | 92.26525 |
| PC 3 | 0.1976151 | 4.940378 | 97.20563 |
| PC 4 | 0.1117747 | 2.794369 | 100.00000 |

**Supplementary info S4** – All results from morphological analyses including data subsets (ie. All offspring born, only offspring included in postnatal treatments, only offspring included in enclosure assays). Interaction p values calculated using Type III Wald Chi-square tests.

|  |  | Fixed effects | b | SE | T | *p* | CI (95%) |
| --- | --- | --- | --- | --- | --- | --- | --- |
| a) Mass at birth | N = 113 | Intercept | *1.38* | *0.03* | *44.92* | *<0.001* | *1.32, 1.44* |
|  |  | Prenatal GC treatment (+GC) | -0.09 | 0.04 | -2.14 | **0.04*** | -0.17, -0.01 |
|  | N = 96 | Intercept | 1.38 | 0.03 | 42.91 | <0.001 | -0.17, 0.00 |
|  |  | Prenatal GC treatment (+GC) | -0.09 | 0.04 | -1.96 | **0.05*** | -1.17, 0.00 |
|  | N = 50 | Intercept | 1.33 | 0.05 | 28.11 | <0.001 | 1.24, 1.43 |
|  |  | Prenatal GC treatment (+GC) | -0.06 | 0.07 | -0.86 | 0.39 | -0.20, 0.08 |
| b) Condition at birth | N = 113 | *Intercept* | *-1.03* | *0.29* | *-3.62* | *<0.001* | *-1.60, -0.47* |
|  |  | Prenatal GC treatment (+GC) | -0.05 | 0.03 | -1.57 | 0.19 | -0.11, 0.01 |
|  |  | SVL | 0.66 | 0.08 | 8.47 | **<0.001***** | 0.50, 0.81 |
|  | N = 96 | Intercept | -1.25 | 0.32 | -3.91 | <0.001 | -1.89, -0.62 |
|  |  | Prenatal GC treatment (+GC) | -0.04 | 0.03 | -1.44 | 0.15 | -0.11, 0.02 |
|  |  | SVL | 0.72 | 0.09 | 8.25 | **<0.001***** | 0.55, 0.89 |
|  | N = 50 | Intercept | -1.50 | 0.42 | -3.55 | <0.001 | -2.36, -0.65 |
|  |  | Prenatal GC treatment (+GC) | -0.001 | 0.04 | 0.02 | 0.98 | -0.09, 0.09 |
|  |  | SVL | 0.78 | 0.12 | 6.72 | **<0.001***** | 0.55, 1.01 |
| c) Mass | N = 88 | Intercept | 1.83 | 0.21 | 8.75 | <0.001 | 1.42, 2.25 |
|  |  | Prenatal GC treatment (+GC) | -0.13 | 0.06 | -2.00 | **0.05*** | -0.26, 0.00 |
|  |  | Social treatment (solitary) | 0.01 | 0.05 | 0.25 | 0.80 | -0.09, 0.12 |
|  |  | Day | 0.02 | 0.0006 | 23.87 | <0.001 | 0.01, 0.02 |
|  |  | Maternal dose number | -0.02 | 0.009 | -1.93 | **0.05*** | -0.04, 0.00 |
|  | *Interactions removed: Prenatal GC x social treatment X^2^ = 0.02, p = 0.90; Prenatal GC x Mat Dose X^2^ = 0.01, p = 0.92* | | | | | | |
|  | N = 44 | Intercept | 1.48 | 0.08 | 18.64 | <0.001 | 1.32, 1.63 |
|  |  | Prenatal GC treatment (+GC) | -0.26 | 0.09 | -2.99 | **0.003**** | -0.43, -0.09 |
|  |  | Social treatment (solitary) | -0.03 | 0.08 | -0.39 | 0.70 | -0.19, 0.13 |
|  |  | Day | 0.02 | 0.0007 | 20.55 | **<0.001** | 0.01, 0.02 |
|  | *Interactions removed: Prenatal GC x social treatment X^2^ = 0.21, p = 0.65; Prenatal GC x Mat Dose X^2^ = 1.23, p = 0.27*  *Other terms removed: Mat Dose T133 = -0.03, p = 0.97* | | | | | | |
| d) Condition | N = 96 | Intercept | -2.79 | 0.30 | -9.30 | <0.001 | -3.38, -2.20 |
|  |  | Prenatal GC treatment (+GC) | -0.09 | 0.04 | -2.48 | **0.01*** | -0.16, -0.02 |
|  |  | Social treatment (solitary) | 0.05 | 0.03 | 1.69 | 0.09 | -0.01, 0.12 |
|  |  | Day | 0.003 | 0.001 | 3.57 | **<0.001***** | 0.00, 0.01 |
|  |  | SVL | 1.14 | 0.08 | 14.23 | **<0.001***** | 0.98, 1.30 |
|  | *Interactions removed: Prenatal GC x social treatment X^2^ = 1.19, p = 0.28; Prenatal GC x Mat Dose X^2^ = 1.77, p = 0.18*  *Other terms removed: Mat Dose T210 = 0.42, p = 0.68* | | | | | | |
|  | N = 50 | Intercept | -2.99 | 0.51 | -5.86 | <0.001 | -4.00, -1.98 |
|  |  | Prenatal GC treatment (+GC) | -1.08 | 0.43 | -2.48 | **0.01*** | -1.93, -0.22 |
|  |  | Social treatment (solitary) | 0.08 | 0.05 | 1.69 | 0.09 | -0.01, 0.17 |
|  |  | Day | 0.003 | 0.001 | 1.99 | **0.05*** | 0.00, 0.01 |
|  |  | SVL | 1.25 | 0.11 | 11.63 | **<0.001***** | 1.03, 1.46 |
|  |  | Maternal dose number | -0.007 | 0.01 | -0.57 | 0.57 | -0.03, 0.02 |
|  |  | Maternal dose number*Prenatal GC treatment (+GC) | 0.04 | 0.02 | 2.07 | **0.04*** | 0.00, 0.08 |
|  | *Interactions removed: Prenatal GC x social treatment X^2^ = 0.19, p = 0.66* | | | | | | |

**Supplementary info S5 – Social network visualisations**

Figure S5.1 Social networks in enclosures 1-5. Each circle represents an individual, circle size is proportional to the number of unique interactions with conspecifics that an individual has, and the thickness of the ties between circles represents the strength of interactions between two individuals. Mothers and offspring are identifiable by naming convention (i.e. Mother E1_1, Offspring E1_1-1, E1_1-2).

**Supplementary info S6 –** Effects of maternal dose number, mass at release

- ***Mass at release*** had no effect on our metric of exploration (T38 = 0.22, P = 0.83). Mass at release (day 21) had no effect on any social network metric (number of interactions Z 39 = -0.18, P = 0.85; network score (strength of interactions) T39 = -1.15, P = 0.26; mother-offspring association T39 = 1.52, P = 0.14. Mass at release (day 21) had no effect on exploration (Z 39 = -1.33, P = 0.19) or dispersal (Z39 = -1.77, P = 0.08).
- We tested potential ***interactive effects of maternal dose*prenatal GC treatment***, with the expectation that treatment duration may have a stronger effect in GC-treated females.
  - No effect on birth mass X2 = 0.01, p = 0.92
  - No effect on condition at birth (mass controlling for SVL) X2 = 0.0004, p = 0.98
  - Mass over time (days 10 – recapture) X2 = 0.01, p = 0.92
  - Condition over time (days 10 – recapture) X2 = 1.77, p = 0.18
  - Growth rate (X2 = 0.30, p = 0.59)
  - Exploration (X2 = 0.78, p = 0.38)
  - Days sighted (X2 = 0.15, p = 0.90)
  - Whether offspring explored dispersal side (X2 = 0.88, p = 0.34)
  - Dispersal (X2 = 0.17, p = 0.86)
  - Number of nodes in social network (offspring: X2 = 0.28, p = 0.59; adults: X2 = 0.01, p = 0.91)
  - Network score (strength of associations) (offspring: X2 = 0.23, p = 0.63; adults: X2 = 0.21, p = 0.65)
  - Association with mother (X2 = 1.78, p = 0.18)
  - Association with sibling (X2 = 0.01, p = 0.90)
- ***Maternal dose number*** (captivity duration) had no effect on birth mass (T107 = -0.77, P = 0.44), condition at birth (T106 = -0.48, P = 0.63), condition over time (T210 = 0.42, P = 0.67), growth rate (T35 = 1.48, P = 0.15), exploration (T37 = -0.32, P = 0.75), days sighted (X2 = 0.12, P = 0.72), dispersal (Z= -1.42, P = 0.16), number of social network nodes (Adults: Z = -1.85, P = 0.06), social network association strength (offspring: T38 = 0.09, P = 0.93; adults: T20 = 0.68, P = 0.50), association with mothers (T38 = 0.41, P = 0.68), association with siblings (T16 = 0.63, P = 0.54).
  Trend for mothers in captivity longer (higher dose number) to have smaller offspring (T211 = -1.93, P = 0.05, CI 95% -0.04, 0.00), for offspring to be less likely to explore dispersal side (Z = -1.94, P = 0.05), for offspring to have fewer social network nodes (Z = -2.21, P = 0.03).
